# Long-term clinical effects of an inhaler guidance DVD in patients with bronchial asthma

**DOI:** 10.20407/fmj.2020-012

**Published:** 2020-11-13

**Authors:** Takazumi Yoshida, Rieko Kondo, Takahiko Horiguchi

**Affiliations:** Department of Internal Medicine (Respiratory Medicine II), Fujita Health University, School of Medicine, Nagoya, Aichi, Japan

**Keywords:** Frequency of aggravation, Inhalation guidance DVD, Inhaled steroids, Long-term effects

## Abstract

**Objectives:**

Erroneous use of inhalers is a serious problem. Given the multitude of devices currently available, it can be difficult to convey the correct methods for their efficient use to patients. We previously generated an educational DVD that visually and audibly explains the proper use of all inhaler types available in Japan to provide inhalation guidance to patients. Herein, we report the 1-year follow-up of patients who received or did not receive the DVD guidance.

**Methods:**

Sixty-nine bronchial asthma patients undergoing outpatient treatment who received inhalation guidance from a pharmacist using a standard package insert were randomly allocated to a DVD group (*n*=35) or a no-DVD group (*n*=34). Their current oral or inhalant drug regimens were unchanged. Various parameters were measured 12 months later. Frequencies of aggravation during the 12-month period were also determined.

**Results:**

Compared with the no-DVD group, there were significant improvements in asthma control test scores, forced vital capacity, FEV1, impulse oscillometry, resonant frequency, induced sputum eosinophil count, and FeNO in the DVD group after 12 months. Pulmonary function and inflammation parameters improved significantly with the use of the instructive DVD in addition to the package inserts. The frequency of asthma aggravation significantly decreased in the DVD group during the 12-month study period, likely because inhalation procedures were performed accurately.

**Conclusions:**

A DVD that provides accurate inhalation guidance enhances the quality of life of asthma patients and has substantial clinical ramifications. Thus, this tool would be beneficial for patients in Japan and worldwide.

## Introduction

In conjunction with elucidation of the pathology of bronchial asthma and chronic obstructive pulmonary disease (COPD) in recent years, the types of available asthma inhaler devices have increased. As a result, both patients and medical professionals often do not fully understand the current situation of inhaler use. The incorrect use of such devices is regarded as a serious problem in numerous countries.^1^ There have even been reports in which drug failure has resulted in disease misdiagnosis, leading to a step-up in treatment.^2^ In an effort to address these issues, in collaboration with the Environmental Restoration and Conservation Agency in Japan, we generated a digital versatile disc (DVD) and user manual titled “Let’s learn the correct way to use inhalers”.^3^ Therein, the correct methods are described for using 12 types of inhalers being sold in Japan as of 2015 (http://www.erca.go.jp).^4^ Associated data including clinical effects 4 weeks after viewing the DVD have been previously published.^4^ These investigations were continued in a larger number of patients in an effort to evaluate the long-term efficacy, and the current study reports the clinical effects after 12 months.

## Methods

**Figure 1:**
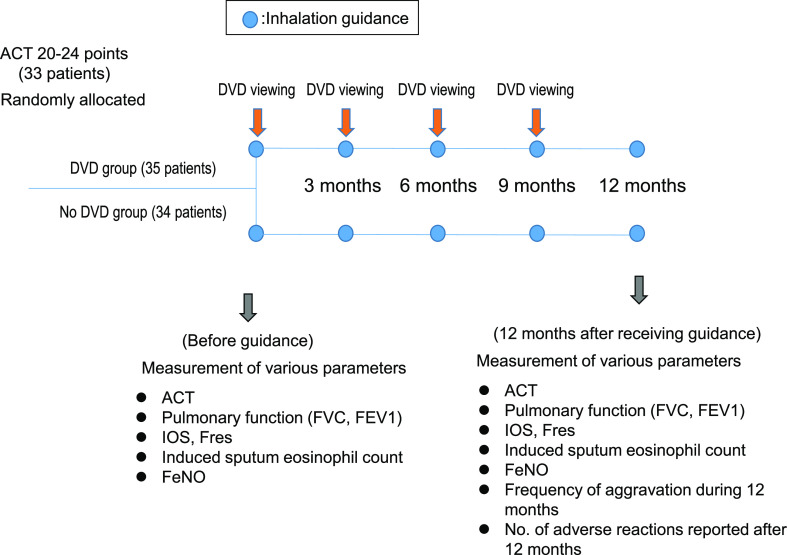
Study design

The current study included 69 bronchial asthma patients undergoing outpatient treatment at a single facility from April 2015 to March 2018. The mean patient age was 61.43±16.75 years (range 32–89 years); there were 32 males and 37 females. Forty patients were at “treatment step 3” as described in JGL2018,^5^ and 29 patients were at “treatment step 4”. There were no seizures during the 1-month observation period, and all patient asthma control test (ACT) scores were between 20 and 24 points. The ACT is a patient-reported measure based on Global Initiative for Asthma (GINA) for classification. This measure includes five items: 1) daytime asthma-related symptoms, 2) limitations of activities, 3) nocturnal symptoms and awakenings, 4) need for rescue treatments, and 5) an overall rating of asthma control over the course of 4 weeks. Individuals were asked to respond using a 5-point scale. The status was defined as: Not well-controlled (ACT score <20), somewhat controlled (ACT score ≥20), and completely controlled (ACT score=25). Written informed consent was provided by all patients via a designated form prescribed by the Fujita Health University Medical Research Ethical Review Board (form 15-334). Figure 1 shows the study design. All patients were undergoing treatment with inhaled corticosteroids and a long-acting β2 agonist. At the time that they commenced taking the drug, they received inhalation guidance from a pharmacist using the standard package inserts and practice devices. No changes were made to their existing oral or inhalant drug regimens. The patients were randomly allocated to a DVD group (*n*=35) and a no-DVD group (*n*=34). Patients in the DVD group were shown a DVD and received inhalation guidance from a pharmacist at the initial outpatient visit, as well as once every 3 months. The DVD was provided to the patients at the initial guidance session so that they had the option of watching it at home as required. Patients in the no-DVD group only received inhalation guidance from the pharmacist at the initial visit and once every 3 months thereafter. The following parameters were measured in all patients: ACT, forced vital capacity (FVC), forced expiratory volume in 1 second (FEV1), impulse oscillometry (IOS), resonant frequency (Fres), induced sputum eosinophil count, and fractional exhaled nitric oxide (FeNO). Spirometry using a CHESTAC8800D device (CHEST Corporation, Tokyo, Japan) was used to acquire the FVC and FEV1 measurements, and a Master Screen-IOS (Jaeger, Hoechberg, Germany) was used to measure Fres while breathing at rest for 30 seconds. To obtain induced sputum eosinophil counts, the patients were instructed to inhale a 5% hypertonic saline solution for 20 minutes continuously. Their sputum was then collected and analyzed after cell fractionation. FeNO was measured using a NiOX instrument (Aerocrine, Stockholm, Sweden). “Acute aggravation” was defined as the presence of at least one of the following: symptoms persisting for 3 days or more; use of short acting beta agonist (SABA) more than twice per day; administration of oral or intravenous steroids. The frequency of aggravation 12 months before the intervention and 12 months after the intervention was compared between the two groups. The data were analyzed via *t*-tests, and *p*<0.05 was deemed to indicate statistical significance. All statistical analyses were performed using StatView J-5.0 software (SAS Institute Inc., Cary, NC, USA).

## Results

**Figure 2:**
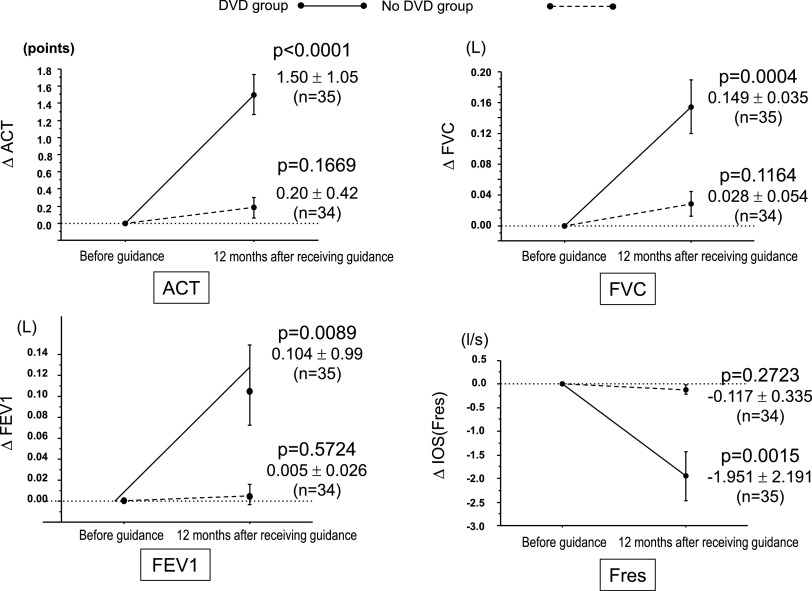
Changes in respiratory function parameters 12 months after undergoing inhalation guidance using a DVD.

**Figure 3:**
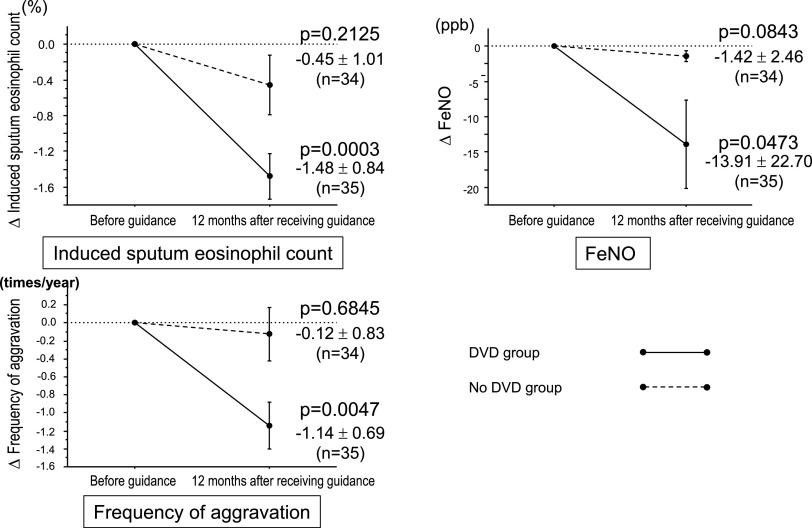
Changes in respiratory function parameters 12 months after undergoing inhalation guidance using a DVD.

Baseline measurements in the DVD group and the no-DVD group are presented in Table 1. There were no significant differences in these baseline parameters between the two groups. In the DVD group, the measurements of all seven experimental parameters acquired at the 12-month time point differed significantly from the corresponding baseline measurements (Figures 2 and 3; ACT *p*<0.0001, FVC *p*=0.0004, FEV1 *p*=0.0089, Fres *p*=0.0015, induced sputum eosinophil count *p*=0.0003, FeNO *p*=0.0473, and frequency of aggravation during a 12-month period *p*=0.0047). None of the corresponding comparisons between baseline values and the 12-month time point values differed significantly in the no-DVD group. In both groups, no adverse events occurred during the study period.

## Discussion

The importance of inhalation guidance is stated in the 2018 GINA report^6^ and in the The National Assessment of Educational Progress (NAEP) report.^7^ The erroneous use of inhalers has been reported as a serious problem worldwide.^8,9^ In a recent study using St. George’s Respiratory Questionnaire scores, asthmatic symptoms and aggravation frequency had evidently improved as a result of thorough inhalation guidance being provided by community pharmacists.^10^ Thus, in collaboration with the Environmental Restoration and Conservation Agency we produced a DVD and user manual^3^ titled “Let’s learn the correct way to use inhalers”. We found that the lung function was significantly higher in the group that was given inhalation instruction 3 months after the first instance of inhalation instruction. In the group not given inhalation instruction, the lung function was lower. Continuity of inhalation instruction is generally considered important.^11^ However, medical staff must devote a certain amount of time to be trained in how to provide proper inhalation instruction, and this time commitment was found to be problematic. Trial and error was repeated, and it was necessary to develop a tool that would help medical staff perform this function. Therefore, a DVD was created that demonstrated the correct methods for using 12 types of inhalers being sold in Japan as of 2015. The DVD is being distributed throughout Japan free of charge, and can also be viewed on the Environmental Restoration and Conservation Agency website (https://www.erca.go.jp/yobou/zensoku/basic/adult/control/inhalers/method01.html). A poster given to the patient shows a Quick Response (QR) code that allows users to read the device operation instructions on their smartphones and/or tablet computers. The DVD educates viewers on the relevant techniques, both visually and audibly. With these educational resources on hand, patients can review the steps if they forget them after returning home. Elderly patients can review the correct procedures by watching the DVD at home or at care facilities, together with friends, family members, or carers. This makes the DVD extremely useful and effective.

In a previously reported study investigating the clinical effects associated with the same DVD learning aid,^4^ at the 4-week time point there were significant improvements in ACT, inhalation techniques, FVC, FEV1, IOS, Fres, and induced sputum eosinophil count in the DVD group compared with the no-DVD group. It is believed that by watching the DVD, the patients were able to learn the inhalation procedures both visually and audibly. This enabled them to use the inhalers more accurately, facilitate the delivery of appropriate amounts of drugs to the trachea, and obtain the desired anti-inflammatory effects.

In the current study, the effects of the educational intervention were investigated 12 months after its implementation, including the frequency of aggravation. The suppression of airway inflammation and maintaining normal respiratory function that is free of asthma symptoms are primary management goals. It is also important to prevent symptom aggravation and avoid future risks. The frequency of aggravation during the 12-month study period was significantly lower in the DVD group. It was previously reported that inhalation guidance offered every 3 months was associated with improved clinical effects.^11^ The results of the current study suggest that this could be further improved by having patients watch the instructive DVD in addition to such guidance.

The DVD makes it possible to convey all of the necessary inhaler instructions to patients without the worry of a clinician omitting an important point. Use of the DVD for inhalation instruction, especially in areas with no respiratory allergy specialists or pharmacists who are experts in inhalation guidance, may help to eliminate disparities in inhalation guidance. In Japan and elsewhere, it is important to improve the ways in which clinicians accurately convey inhalation instruction to patients to achieve better asthma control.

## Figures and Tables

**Table1:** Baseline measurements

	DVD group (n=35)	No DVD group (n=34)	
Age	61.75±15.25	60.50±18.8	n.s.
Sex (male/female)	16/19	16/18	n.s.
History of smoking (never/ex)	11/24	10/24	n.s.
Treatment step (3/4)	20/15	20/14	n.s.
ACT	21.76±1.87	21.91±1.31	n.s.
FVC (L)	2.98±0.99	2.89±1.11	n.s.
FEV1 (L)	2.15±0.92	2.34±0.77	n.s.
IOS(Fres) 1/s	16.89±7.12	17.21±6.98	n.s.
Induced sputum eosinophil count (%)	2.52±1.30	2.50±1.70	n.s.
FeNO (ppb)	31.28±39.2	30.56±35.6	n.s.
Frequency of aggravation	2.42±0.53	2.12±0.83	n.s.
